# Minimal-Access Coronary Revascularization: Past, Present, and Future

**DOI:** 10.3390/jcdd10080326

**Published:** 2023-07-31

**Authors:** Rushmi Purmessur, Tharushi Wijesena, Jason Ali

**Affiliations:** Department of Cardiothoracic Surgery, Royal Papworth Hospital NHS Foundation Trust, Cambridge CB2 0AY, UK; rtw27@cam.ac.uk (T.W.); jason.ali@nhs.net (J.A.)

**Keywords:** minimal access, coronary artery, revascularisation, MIDCAB, TECAB, HCR

## Abstract

Minimal-access cardiac surgery appears to be the future. It is increasingly desired by cardiologists and demanded by patients who perceive superiority. Minimal-access coronary artery revascularisation has been increasingly adopted throughout the world. Here, we review the history of minimal-access coronary revascularization and see that it is almost as old as the history of cardiac surgery. Modern minimal-access coronary revascularization takes a variety of forms—namely minimal-access direct coronary artery bypass grafting (MIDCAB), hybrid coronary revascularisation (HCR), and totally endoscopic coronary artery bypass grafting (TECAB). It is noteworthy that there is significant variation in the nomenclature and approaches for minimal-access coronary surgery, and this truly presents a challenge for comparing the different methods. However, these approaches are increasing in frequency, and proponents demonstrate clear advantages for their patients. The challenge that remains, as for all areas of surgery, is demonstrating the superiority of these techniques over tried and tested open techniques, which is very difficult. There is a paucity of randomised controlled trials to help answer this question, and the future of minimal-access coronary revascularisation, to some extent, is dependent on such trials. Thankfully, some are underway, and the results are eagerly anticipated.

## 1. Introduction

Coronary artery revascularisation has become the most common cardiac surgical procedure performed worldwide. Interestingly, coronary artery bypass grafting as we know it finds its roots in minimal-access approaches when the first coronary artery bypass grafts were performed by left anterolateral mini-thoracotomies without cardiopulmonary bypass (CPB). 

Minimal-access cardiac surgery is becoming fashionable, and as more surgeons adopt minimal-access techniques, there is a need for all cardiac surgeons to be aware of what is becoming available so that they can offer their patients the best treatment. However, many remain sceptical about minimal-access techniques in cardiac surgery and highlight the concerns surrounding these approaches and, in many cases, there remains a paucity of evidence demonstrating clear benefits over traditional median sternotomy, which remains the commonest approach to performing cardiac surgery.

In this review, we aim to focus on the history of minimal-access coronary artery revascularisation and move to discuss the patient selection and the techniques and evidence supporting the common minimal-access approaches to coronary artery revascularisation. These approaches include minimally invasive direct coronary artery bypass grafting (MIDCAB), totally endoscopic coronary artery bypass grafting (TECAB), and hybrid coronary revascularisation (HCR).

## 2. Materials and Methods

A search was conducted in the PUBMED online database using the following search terms: “minimally invasive coronary artery revascularisation”, “minimal access coronary artery revascularisation”, “minimally invasive cardiac surgery coronary artery bypass grafting”, “robotic assisted cardiac surgery”, “endoscopic cardiac surgery”, “robotic assisted thoracoscopic surgery coronary artery bypass graft surgery” “minimally invasive direct coronary artery bypass graft”, “total endoscopic coronary artery bypass grafting”, and “hybrid coronary revascularisation”. The search was limited to reviews, meta-analyses, and randomised controlled trials (RCT) from January 1997 to December 2022.

## 3. History of Coronary Artery Bypass Grafting

The history and evolution of coronary artery bypass grafting (CABG) has been rife with successes and failures. The first CABG was performed by Alexis Carrel [1,2] in 1910 in dogs before the advent of coronary angiography or cardiopulmonary bypass (CPB). In the 1930s, John Gibbon invented the CPB machine, which revolutionised cardiac surgery [3]. Later, in 1946, Arthur Vineburg [4,5] pioneered the Vineburg technique, whereby he implanted the left internal mammary artery (LIMA) directly onto the left ventricular myocardium, which led to symptomatic relief of angina and was shown to still provide good cardiac function 30 years later [6]. The first LIMA to left anterior descending artery (LAD) anastomosis using a non-suture technique with tantalum rings appeared a few years later, in 1952, when Demikhov showed graft patency in the LIMA to LAD anastomosis at 2 years, a practice that was also adopted by others in Canada [7] and the US [8]. In 1956, Charles Bailey successfully performed coronary artery endarterectomies as a way to treat coronary artery atherosclerosis [9].

The issue, however, remained that the arteries could not be imaged and, therefore, the uncertainty of which arteries caused the symptoms persisted. This changed in 1958, when Mason Sones [10] inadvertently performed the first coronary angiogram by accidentally injecting dye in the right coronary artery when attempting to image a patient with rheumatic heart disease. He then went on to further develop coronary angiography—an achievement that changed the history of cardiovascular medicine.

In 1962, Sabiston [7] performed the first hand-sewn coronary anastomosis by suturing a saphenous vein graft to the right coronary artery—a procedure performed without CPB—but it was not reported until 1974. Garrett [11] and DeBakey in Houston also performed hand-sutured coronary anastomoses in 1964 but did not report it until 1973 when the grafts remained patent 7 years later. Kolessov, on the other hand, reported his first few CABGs with hand-sutured coronary anastomoses early in 1967—all procedures were performed without CPB in 1964 [12]. Despite being heavily involved in pioneering CPB as an artificial circulation for open-heart surgery, Kolessov was a great proponent of off-pump CABG owing to the large inflammatory response that CPB generated at the time. It was not till 1968 that Green [13] in New York performed the first hand-sutured LIMA to LAD anastomosis, which has since become the cornerstone of coronary artery revascularisation.

Moving on to the late 1960s and early 1970s, Favoloro [14] in the Cleveland Clinic really pushed forward the use of saphenous vein grafts as a conduit during coronary artery revascularisation. However, it was realised early on that owing to intimal [15,16] and medial thickening and graft thrombosis secondary to intimal hyperplasia and premature atherosclerosis of the vessel, saphenous vein grafts were prone to stenosis and occlusion. Carpentier [17] started using radial arteries as a conduit—the early experience of which was not as successful as it is today. The introduction of the no-touch technique of vein and radial artery harvesting in the early 1990s by Acar [18,19], as well as the use of vasodilators for radial artery grafts significantly, improved the long-term patency of veins and radial arteries as conduits for coronary artery revascularisation and revived the interest in using radial arteries as a conduit. It was only in the 1980s that the LIMA to LAD anastomosis was proven beyond doubt to have a prognostic benefit when Loop et al. in Cleveland clinic reported their ten-year outcomes [20].

Meanwhile, in the late 1970s, cardiologists started developing percutaneous catheter-based interventions (PCIs), initially with balloon angioplasty [21] but progressing to stenting and then more recently using drug-eluting stents to overcome the complications of in-stent restenosis observed in early versions of bare metal stents. PCIs had the overwhelming advantage of being less painful, with a shorter recovery and smaller risk of stroke.

To potentially challenge these advantages of PCIs but still obtain the higher survival rates that surgery conferred, the surgical community began to turn to minimal-access coronary surgery. In the mid-1990s, Calafiore reported isolated LIMA to LAD anastomoses performed through an anterior thoracotomy [22]. At the same, Peters described what was later renamed “The HeartPort technique”, after the company Heartport developed a three-lumen catheter to be placed through the groin into the aorta, where one lumen would endovascularly occlude the aorta, the second lumen would be used to deliver cardioplegia, and the third lumen used as a root vent [23]. This has since progressed more recently to coronary revascularisation performed with fully thoracoscopic and robotic methods, with the first TECABG being performed by Loulmet [24] in 1998. Now, many centres around the world have introduced minimal-access coronary surgery with varying permutations, from mini-thoracotomy off-pump LIMA to LAD anastomosis in MIDCAB to fully robotic complete revascularisation.

**Figure 1:**
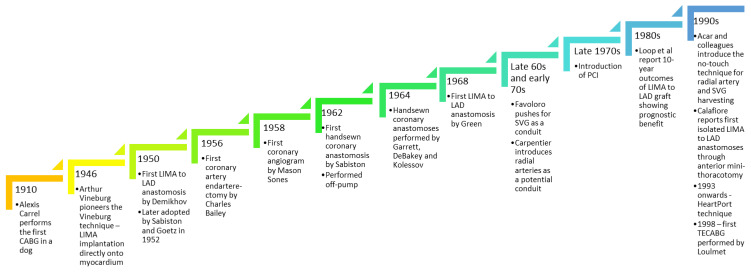
History of minimal-access coronary artery surgery.

A timeline summarising major events in coronary artery revascularisation has been summarised in Figure 1.

## 4. Minimal-Access Coronary Revascularisation—International Guidelines Perspective

The 2018 EACTS/ESC guidelines on myocardial revascularisation do not make any formal recommendation regarding minimal-access surgery, but they do mention that it is an attractive alternative to conventional approaches for CABG surgery [25].

The guidelines highlight HCR to be an appealing management strategy, whether performed sequentially, i.e., minimal-access LIMA to LAD anastomosis followed by PCIs to the non-LAD vessels in another setting or performed in a hybrid theatre in one session, quoting the POL-MIDES RCT [26,27] where, in a small group of 200 patients, conventional surgery and HCR had similar outcomes at 5 years. Of course, it is important to consider if 5-year outcomes are long-term enough to justify the non-inferiority of HCR compared to more traditional approaches.

Similarly, the 2021 ACC/AHA/SCAI guidelines on coronary artery revascularisation comment that the role of HCR remains unclear and do not make any formal recommendation as to when it can or should be used [28]. They, however, do not comment on any other method of minimal-access coronary artery revascularisation surgery.

## 5. Patient Selection and Rationale for Minimal-Access Coronary Intervention

The surgical indication for minimal-access coronary revascularisation remains unclear in the literature. Some small studies in the early 1990s and 2000s [29] report the use of minimal-access coronary artery revascularisation surgery for patients with isolated coronary artery disease, isolated LAD lesions, or proximal right coronary artery disease. However, the conduct of minimal-access coronary artery revascularisation surgery, from patient selection, the use of CPB or lack thereof, to even conduit selection for different lesion sets, is too varied to make any reasonable conclusion as to where the actual benefit of minimal-access CABG lies. The advantage of minimal-access coronary artery revascularisation presumably is more apparent in patients with uncontrolled diabetes or multiple co-morbidities, which confer a higher risk of sternal wound non-healing, breakdown, and infection. In addition, in those performing minimal-access CABG off-pump, there are added benefits such as a reduced stroke [30] rate from the absence of aortic manipulation and cross-clamping, decreased inflammatory [31] response from the bypass circuit leading to lower rates of acute kidney injury [32], and fewer blood transfusions. Moreover, if a mini-thoracotomy is performed, no bone healing is required post-operatively, allowing patients to return to their normal lifestyle more rapidly. With the smaller incisions, patients can be extubated faster and there are fewer complications of respiratory failure. Diegeler et al. in a small prospective trial suggested that after post-operative day 4, MIDCABG had lower rates of pain compared to conventional CABG [33].

There are some patient features that are favourable for minimal-access CABG, including being slim and having a thin, tubular, and vertically positioned heart. LAD lesions that tend themselves to minimal-access surgery are those with a non-calcified distal segment (approximately 2–4 cm distal to the second diagonal branch), those with an arterial diameter greater than 1.75 mm, and total occlusion of the LAD with good collateral circulation [34].

However, it should be kept in mind that while some centres consider multivessel disease a contraindication to minimal-access coronary revascularisation, others regularly perform multi-vessel grafting using minimal-access methods.

## 6. Contraindications to Minimal-Access Coronary Revascularisation

The only absolute contraindications to using a minimal-access approach are an occluded left subclavian artery, which prevents the use of the LIMA, particularly in hybrid procedures where the benefit is a LIMA to LAD anastomoses, and patients in cardiogenic shock require emergent LAD revascularisation due to the longer LIMA harvesting time and the longer setup time for certain methods of minimal-access surgery [35].

Relative contraindications depend on the surgeon and their experience and the institution. These include extreme obesity, which makes access and LIMA harvesting more challenging, deep intramural and calcified LAD grafting sites, which are more challenging to identify in a minimal-access setting, previous thoracotomy, re-do surgeries, and the presence of dense adhesions, which all restrict exposure and distort the anatomy, and the presence of severe pulmonary hypertension with a large left ventricle, making the minimal-access approach higher risk and more technically challenging. While some co-morbidities would lend themselves for patients to have better outcomes with minimal-access surgery, they can often be prohibitive as well. For example, patients who are unable to tolerate single lung ventilation might not be able to undergo minimal-access surgery despite potentially benefitting greatly from the early extubation and reduced rates of respiratory failure observed with minimal-access surgical coronary revascularisations. In addition, the presence of significant peripheral vascular disease may mean that going onto peripheral cardiopulmonary bypass via the femoral vessels may not be an option intra-operatively if cardiopulmonary bypass were required—such patients should be treated with caution [34,35,36].

## 7. Techniques of Minimal-Access Coronary Artery Revascularisation

In this section, we will describe the common approaches to minimal-access coronary revascularisation surgery. We will describe patient positioning, as well as some technical considerations. The advantages and disadvantages of the different techniques are summarised in Table 1.

### 7.1. MIDCABG

#### 7.1.1. Description

MIDCABG has been described using multiple methods and approaches in the literature. The first few descriptions of MIDCABG surgery were purely describing a LIMA–LAD anastomosis. The surgical technique has now evolved to include multivessel grafting. While most commonly performed via a left anterior mini-thoracotomy in the fourth intercostal space in the infra-mammary fold underneath the nipple with 2/3 of the incision being medial and 1/3 lateral to the nipple [36], some centres also describe accessing the chest via an upper partial sternotomy or inferior partial sternotomy. MIDCABG started as being a way of performing open-heart coronary artery revascularisation with no sternotomy but has gradually evolved to using endoscopic instruments to facilitate the process.

#### 7.1.2. Positioning and Monitoring

The patient should be placed in an anterolateral decubitus position with the left chest and left buttock elevated by approximately 20–30 degrees using a bolster if the approach is to be through a left anterolateral mini-thoracotomy. If a partial superior or partial inferior sternotomy is to be used, then the patient can be in a supine position. The arms of the patient should be tucked at the sides. Regardless of whether peripheral CPB is used routinely or as a safety measure for emergent situations, the left groin should be prepared and draped. A guidewire is sometimes inserted under ultrasound guidance into the left femoral artery prior to prepping and draping to facilitate the emergent institution of CPB if required. External pacing and defibrillator pads, as well as warming blankets, should also be routinely placed and connected. Each institution will have its own monitoring protocols. However, it is advisable to use a pulmonary artery catheter in patients with a left ventricular ejection fraction of <30%, ECG monitoring for ischaemia, urinary bladder catheterisation, and temperature probe insertion. Transoesophageal echocardiography is used in patients with poor ventricular function or who are at higher risk of becoming haemodynamically unstable.

#### 7.1.3. Operative Steps

**Figure 2:**
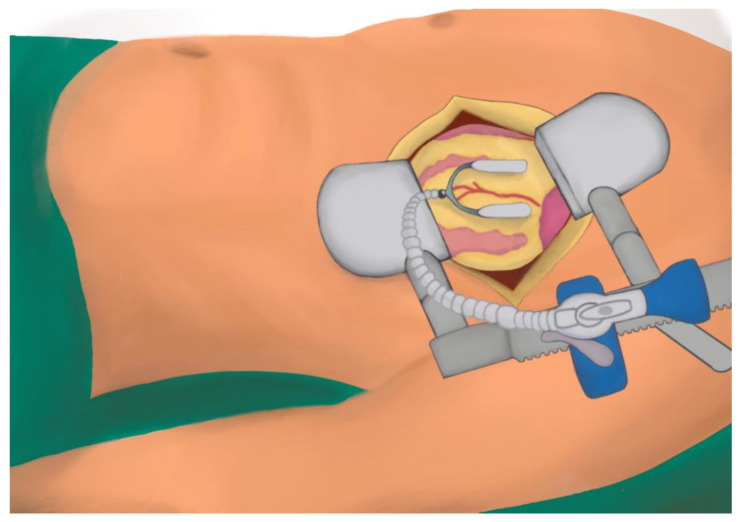
Left thoracotomy for MIDCABG with a heart stabilising device.

The most common approach is through a 5–6 cm left anterolateral muscle-sparing mini-thoracotomy in the fourth or fifth intercostal space, 2–3 cm inferior to the nipple (Figure 2) [36]. One-lung ventilation is used to facilitate exposure. A retractor is used for LIMA harvesting, either skeletonised or pedicled, as per the surgeon’s preference. In cases where bilateral IMAs will be used, bilateral mini-thoracotomies can be performed. After harvesting the LIMA, before dividing, the patient is heparinised, the pericardium opened longitudinally, usually 1–2 fingerbreadths lateral to the LIMA pedicle and suspended with traction sutures, and the LAD is identified. The lateral traction sutures are pulled upward to the upper part of the wound, which rotates the heart, exposing the LAD and facilitating anastomosis [36]. The distal end of the LIMA is then divided and prepared for anastomosis. The edges of the pericardium and selective lung inflation can be used to improve the visualisation of the LAD. A suction stabiliser is used to stabilise the LAD for anastomosis. Either a pledgeted tourniquet can be applied around the LAD proximal to the anastomosis or a soft vascular clamp used to occlude the LAD to allow for a bloodless field. Alternatively, a shunt can also be used. Once anastomosis is performed, the flow can be verified, following the restoration of blood flows through the LAD and removal of the pledgeted tourniquet or vascular clamp, for example using transit time flow measurement. Haemostasis is performed and heparin is reversed. The pericardium is closed around the apex, and a chest drain is inserted into the left pleura. The thoracotomy is then closed as per usual [34,43].

#### 7.1.4. Evidence

Patel et al. published the best evidence on this topic by comparing MIDCABG and PCIs for patients with isolated LAD disease in 2014 [44]. They looked at 13 studies and concluded that both are effective treatments. The PCI has higher rates of need for reintervention for symptom recurrence. Despite having a higher upfront cost, MIDCABG is more cost-effective due to the lower rate of reintervention. There was no significant difference in mortality between both groups.

In 2015, Raja et al. [45], on behalf of the Harefield Cardiac Outcomes Research Group, compared propensity score-matched patients undergoing MIDCABG versus full sternotomy revascularisation for isolated LAD disease, with 143 matched sets. In 2018, they compared the short- and long-term outcomes of MIDCABG versus full sternotomy off-pump LIMA to LAD anastomosis for isolated proximal LAD stenosis [46]. They looked at 668 patients, with 508 patients in the MIDCABG group and 160 patients in the full sternotomy off-pump group. The average operative time was significantly shorter in the full sternotomy group, 141 +/− 12 min in the median sternotomy group versus 177 +/− 32 min in the MIDCABG group, *p* = 0.003. There was no significant difference between both groups in terms of the short-term outcomes. The long-term mortality at a median follow-up of 12.95 +/− 0.45 years was 25% in the full sternotomy off-pump group compared to 22.24% in the MIDCABG group, *p* = 0.64.

A study by Repossini et al. in 2019 [47] looked at 1060 patients undergoing MIDCABG, 646 of which had isolated proximal LAD disease and the rest had multivessel disease managed either with HCR or MIDCABG and optimal medical therapy. The reported survival was 92.1 +/− 4.6% at 5 years and 85.3 +/− 6.3% at 15 years, with an overall perioperative mortality of 0.8%.

Manuel et al. [48] recently published their 20-year outcomes of MIDCABG surgery in patients undergoing LIMA to LAD anastomosis. Their cohort consisted of 271 patients—the overall survival was 91.9%, 84.7%, 71.3%, and 56.5% at 5, 10, 15, and 20 years, respectively, with patients with isolated LAD disease doing significantly better than patients with multivessel disease (*p* = 0.0035). There were no patients who required reintervention on the LAD post-operatively.

Ultimately, there are no robust RCTs comparing MIDCABG and PCIs or MIDCABG and conventional CABG via a median sternotomy, and this presents a gap in the literature.

### 7.2. TECABG/RACABG

#### 7.2.1. Description

TECABG is currently the least invasive form of surgical coronary artery revascularisation. It is performed via a few port sites, occasionally using a remotely controlled robotic system. Robotic-assisted TECABG can be further divided into three surgical techniques: TECABG without CPB, TECABG with CPB, and robotic-assisted LIMA harvest followed by off-pump LIMA to LAD manual anastomosis. Other options also include a video-assisted LIMA harvest, followed by manual LIMA to LAD anastomosis via a small anterior mini-thoracotomy.

#### 7.2.2. Positioning and Monitoring

The position of the patient depends on the approach. If the procedure is performed without robotic assistance and by using video thoracoscopic assistance, the patient is placed in a left lateral decubitus position, 30–60 degrees from the horizontal line with the arm above their head [49]. If robotic-assisted TECABG is performed, the patient is placed supine with the left side elevated to 30 degrees and the left arm tucked in at the side [50,51].

Defibrillation pads are plated on the patient pre-operatively. Monitoring is similar to MIDCABG.

#### 7.2.3. Operative Steps

**Figure 3:**
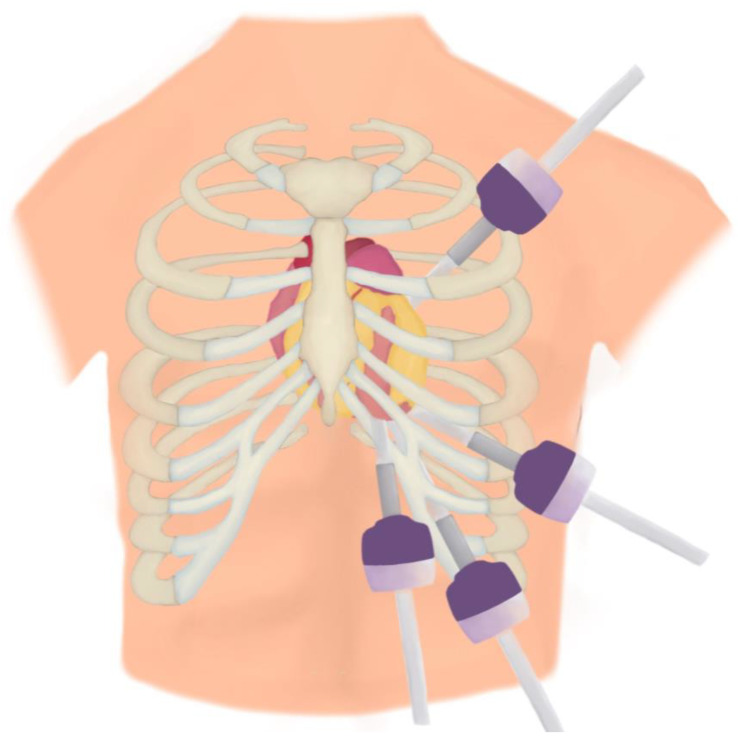
Example of port positions for TECABG—the ports are placed in such a way as to triangulate to the mediastinum, bearing in mind the patient’s body habitus and target vessels.

TECABG is performed with the help of video-assisted thoracoscopy (VATS) or robot-assisted thoracoscopy (RATS). A controlled pneumothorax is induced using carbon dioxide insufflation. This can help create a visual field without one-lung ventilation. However, sometimes, one-lung ventilation may be required, in which case either a double-lumen endotracheal tube or a bronchial blocker can be used. The LIMA and/or RIMA can both be harvested from the left chest using VATS or RATS instruments via ports in the second, third, and fourth intercostal spaces, approximately 2 cm above and below the anterior axillary line, triangulating towards the mediastinum (Figure 3). However, the port placement can be changed depending on the surgeon, patient body habitus, and position of the target vessels. The patient is then heparinised, and the distal ends of the mammary arteries are transected. The pericardium is opened longitudinally, anterior to the left phrenic nerve, and all target vessels are identified and correlated with angiographic findings. Once the target vessels are identified and located, a 3–4 cm port is created directly above the heart close to the midline in the selected intercostal space. CPB can be instituted peripherally via the femoral vessels. A pledgeted purse-string suture for antegrade cardioplegia is inserted in the ascending aorta. After decompression of the right atrium on CPB, an endoscopic transthoracic clamp is inserted in the second right intercostal space in the anterior axillary line and placed across the ascending aorta. Cardioplegia is then delivered in an antegrade fashion via an endoscopically placed vent needle in the proximal ascending aorta. It should be noted in cases of robotic-assisted TECABG, aortic occlusion in on-pump procedures can also be achieved using an endovascular occluding balloon placed and inflated in the ascending aorta under transoesophageal ultrasound guidance. If the procedure is being carried out off-pump, one of the ports is used to insert tissue-stabilising devices. In procedures where only the LIMA harvest is performed using the robotic system, once the LIMA is harvested, the robot is undocked, and the remainder of the procedure is performed, as per MIDCABG. Pericardial stay sutures, epicardial stay sutures, or gentle traction of the emptied heart through a small subxiphoid incision can help visualise the target vessels to facilitate anastomosis. In cases where the remainder of the procedure is performed using the MIDCABG technique, the heart is positioned close to the utility port in the fourth intercostal space close to the midline, and the anastomosis is performed manually. If the robotic system is being used for the distal anastomoses as well, the pericardium is opened, and the robotic arms are used to manipulate the heart and perform the anastomosis, which is described in detail by Bonatti et al. [50] and Lee et al. in 2012 [52]. Y-grafts to the LIMA are generally used for the non-LAD vessels to avoid aortic manipulation. Alternatively, saphenous vein grafts can be sutured to the axillary artery prior to performing the distal anastomosis—they are endoscopically transferred into the left pleural space through an opening next to the LIMA harvest site. After all anastomoses are completed, haemostasis is performed, and the heparin is reversed with protamine. The pericardium is closed using interrupted sutures apart from channels for the LIMA and/or RIMA. Drains are placed in each intra-thoracic cavity. Ports are closed in a standard fashion, in layers [53].

#### 7.2.4. Evidence

There has been no RCT comparing the different types of TECABG and comparing TECABG to conventional CABG.

A systematic review by Cao et al. [54] included 44 studies and a total of 8034 patients and revealed a pooled perioperative mortality rate of 1.7% and 1.0% after off-pump TECAB and robotic-assisted MIDCABG groups, bearing in mind that in the majority of studies, the number of anastomoses was relatively few and patients were relatively young, with a mean age of 60 and good pre-operative left ventricular function, with a mean ejection fraction of more than 55%. Unfortunately, long-term survival was not available due to limited follow-up rates in the included studies.

Although there have been no RCTs comparing outcomes of conventional CABG and TECABG, a study by Kofler et al. 2017 [55] compared 134 propensity score-matched pairs of conventional CABG and robotic TECABG. The primary endpoints were long-term survival and freedom from major adverse cardiac and cerebral events (MACCEs). There was no significant difference in the primary endpoints between both groups at 1, 5, and 10 years. The survival at 1, 5, and 10 years was 99.3%, 96.9%, and 81.3%, respectively, in the robotic group versus 96.3%, 92.2%, and 82.6% in the conventional group, *p* = 0.960. Freedom from MACCE in the robotic group at 1, 5, and 10 years was 97.6%, 96.8%, and 96.8%, respectively, versus 100%, 97.7%, and 92.8% in the conventional group, *p* = 0.790. Of note, robotic TECABG had significantly longer CPB times (robotic 112 +/− 100 min versus conventional 67 +/− 48 min, *p* < 0.001) and cross-clamp times (robotic 68 +/− 54 min versus 38 +/− 27 min in the conventional group, *p* < 0.001.)

A meta-analysis by Leonard et al. in 2018 looking at the outcomes of TECABG including 17 studies and 3721 patients demonstrated that TECABG has acceptably low operative risk [56], but there was a severe dearth of data to confidently recommend TECABG. The pooled operative mortality for 3676 patients was 0.8% with a 95% CI of 0.6–1.2%. The pooled perioperative myocardial infarction event rate for 2556 patients was 2.28% with a 95% CI of 1.7–3%. The overall pooled graft patency rate was 94.8%. The pooled event rate for perioperative stroke was 1.5% with a 95% CI of 1.1–1% with 3353 patients being included.

Gobolos et al. [57] published a systematic review of the clinical outcomes of TECABG over the last 20 years in 2019. The pooled results included 2397 cases and reported a perioperative mortality of 0.8%, with conversion rates of 11.5% and an average surgical time of 291 +/− 57 min. Comparing beating heart TECABG (BH-TECABG) and arrested heart TECABG (AH-TECABG) revealed perioperative mortality of nearly 1% for BH-TECABG and 0.6% for AH-TECABG.

Similarly, a meta-analysis in 2020 by Hammal et al. looking specifically at robotic TECABG and 13 studies and reported that although robotic coronary artery surgery was feasible and certainly an appealing alternative to conventional surgery [58], the level of evidence was too low to make any significant conclusions regarding the benefit of robotic TECABG over conventional CABG in terms of short- and long-term outcomes including perioperative mortality, long-term survival, perioperative stroke, perioperative or late MI, and the rate of revascularisation. The data were too heterogenous to compare pooled event rates between robotic TECABG and conventional CABG.

### 7.3. HCR

#### 7.3.1. Definition

CABG remains the guideline-recommended management option for many patients with multivessel coronary artery disease and has superior long-term survival rates [59]. It has been posited that the superiority of CABG lies with the LIMA to LAD anastomosis [60]. For non-LAD lesions, the PCI potentially confers similar long-term results as saphenous vein grafts. This principle forms the basis of hybrid minimal-access surgery, where LIMA to LAD anastomosis is performed by minimal-access surgery, and the other lesions are managed percutaneously [61].

Hybrid coronary artery revascularisation combines the prognostic benefit of LIMA to LAD anastomosis through minimal-access surgery with the advantages of less pain, decreased length of hospital stays, and the ability to continue dual antiplatelet agents that the PCI confers [62,63]. While it is difficult to specify a target patient population owing to the lack of RCT evidence, the ideal patient would be a high-risk surgical patient with complex or non-stentable LAD lesions who would reap the benefits of LIMA to LAD anastomosis with concurrent stentable non-LAD lesions.

There are three options for HCR: simultaneous revascularisation in a hybrid theatre, surgery followed by PCIs, or PCIs followed by surgery [64,65]. The latter option could follow such an example, where the culprit artery causing an infarct is a non-LAD artery that can be stented, perhaps acutely, with concurrent LAD lesions requiring surgery performed soon thereafter. Whether the surgery is performed via MIDCABG or TECABG is up to the heart team and the institution’s experience.

#### 7.3.2. Evidence

The POL-MIDES (HYBRID) trial in 2014 published by Gasior et al. randomided 200 patients with multivessel disease to undergo either HCR (*n* = 98) or CABG (*n* = 100). The primary endpoint was evaluating the feasibility of HCR, which was defined as the percentage of patients who had a completely hybrid approach with LIMA to LAD followed by PCIs with drug-eluting stents. A total of 93.9% of the patients randomised in the HCR group had a complete hybrid procedure, with 6.1% converting to a standard CABG. The secondary endpoints were post-procedure and angiographic measurements of the graft patency and restenosis rates at 12 months, among others. The mortality from CABG was 2.9% compared to 2% in the HCR group, *p* = 0.1. HCR had a higher HYBRID patency score (free of stenosis/occlusions grafted or ratio of stented arteries to the total number of grafted and stented arteries) at 90% compared to 81% in the CABG group, *p* = 0.01 [26].

In 2019, Ganyukov et al., in the Hybrid coronary REvascularisation Versus Stenting or Surgery (HREVS) prospective randomised safety and efficacy study compared conventional CABG (*n* = 50), HCR (*n* = 52), or multivessel PCIs (*n* = 53), with residual ischaemia as their primary endpoint. They concluded that the percentage of ischaemic myocardium in CABG, HCR, and PCIs was 6.7% (95% CI 4.6–8.8%), 6.4% (95% CI 4.3–8.5%), and 7.9% (95% CI 5.9–9.8%), respectively, *p* = 0.45. The rates of MACCE, one of their secondary endpoints, in CABG, HCR, and PCIs were 12%, 13.4%, and 13.2%, respectively, *p* = 0.83. The main limitation quoted was that the study was severely underpowered and, therefore, not conclusive [66].

In 2020, Esteves et al. published their results of a pilot RCT, the myocardial hybrid revascularization versus coronary artery bypass GraftING (MERGING study) for complex triple-vessel disease comparing HCR to conventional CABG, with 40 patients in the hybrid arm and 20 patients in the conventional CABG arm. They concluded that HCR, while feasible, was associated with higher rates of MACCE defined as all-cause death, stroke, MI, and unplanned revascularisation during the first 2 years as compared to conventional surgery, with a 19.3% MACCE rate at 2 years in the HCR group versus a 5.9% MACCE rate in the conventional group [67].

Guan et al. in 2019 [29] published a meta-analysis comparing other modalities of minimal-access CABG with HCR, which summarised eight observational studies and concluded that HCR was non-inferior to other modalities of minimal-access CABG, in terms of in-hospital mortality, rates of MACCE, shock, perioperative MI, long-term survival, cost, and surgical complications. On the other hand, Nagraj et al. in 2022 concluded in their meta-analysis of twelve observational studies and two RCTs comparing HCR to conventional CABG via a median sternotomy in multivessel coronary artery disease that although feasible, HCR did not have any clear benefits over conventional surgery [68].

Dixon et al. in 2022 [69] published a systematic review and meta-analysis comparing HCR and CABG for multivessel coronary artery disease. Their analysis included 16 studies and concluded that HCR had comparable outcomes to CABG in terms of mid-term survival and rates of MACCE, but patients had a shorter ITU stay and decreased need for blood transfusion. This did not translate into better short-term outcomes of HCR compared to CABG, with a higher incidence of short-term mortality in the HCR group (0.73% versus 0.64% in the CABG group). However, it should be mentioned that the results reported in the HCR group had a much wider confidence interval, indicating a lack of statistical power. Therefore, they were unable to definitely conclude any advantage of HCR over CABG or vice versa. Table 2 summarises some of the major studies regarding minimal access surgical coronary artery revascularisation.

## 8. Nomenclatures

The large variation in the nomenclature used to describe minimal-access surgical techniques for coronary artery revascularization renders the interpretation of the literature challenging and makes comparisons of the different techniques challenging. Just to name a few, the terms MIDCABG, MICS CABG, TECABG, AH-TECABG [80], PA CABG [81], and RACABG have all been used to describe various minimal-access cardiac surgery. Some of these terms are used interchangeably by some authors but considered distinct by others. For example, some papers claim that MIDCABG and MICS CABG are completely different modalities, while others use the terms interchangeably. Similarly, some papers consider TECABG and RACABG to be distinct modalities, while some authors describe, in detail, how they use either VATS or RATS to perform TECABG. We would posit that the standardization of terms is an imperative step to allow robust comparison of minimal-access techniques, be it as compared to each other or conventional CABG.

## 9. Future Perspectives

Minimal-access techniques are gaining popularity in all areas of surgery. The number of cardiac surgical centres with access to minimal-access techniques and surgical robots is continuously increasing. Mitral valve surgery is a particularly hot area for minimal-access surgery—and the publication of the results of the UK Mini Mitral Trial is eagerly awaited. For coronary revascularization, it is important that these new techniques are cautiously adopted and experience is accumulated. For this, large RCTs are required to develop the evidence base to support the use of these techniques and demonstrate conclusively that they are beneficial to patient outcomes. Demonstrating this through trials will be essential to gaining wider adoption of these techniques and for some, the ability to justify the expense of the technology to hospital management.

Thankfully, there are trials ongoing. For example, the Minimally Invasive Coronary Surgery Compared to STernotomy Coronary Artery Bypass Grafting RCT (MIST trial) is an upcoming prospective RCT. It compares the outcomes of minimal-access coronary revascularization to conventional CABG [82]. The primary outcome is the quality of life using the physical function score of the Short Form Health Survey (SF-36) four weeks after surgery. Secondary outcomes include MACCE and target vessel revascularisation at 1 year after surgery, the number of bypass grafts, the percentage of arterial graft use, the use of transfusion intra-operatively and post-operatively, the rates of re-exploration for bleeding, post-operative pain, duration of intubation, length of stay in the intensive care unit, length of hospital stay, the rates of post-operative atrial fibrillation and wound infection, and post-operative angina and quality of life in terms of mental health. It is currently still in the enrolment phase and is projected to be completed primarily in March 2024.

## 10. Conclusions

Minimal-access surgery is becoming increasingly popular and may become the future. It is increasingly demanded by referring cardiologists and also patients who perceive the surgery to be superior. Minimal-access coronary artery revascularization represents a very appealing management approach to coronary artery disease. It incorporates the benefits of surgical revascularization with some of the advantages of off-pump surgery and PCIs with less pain, shorter hospital stays, earlier mobilization, and earlier return to work for patients. However, the challenge is to ensure that the benefits of surgical revascularization with complete revascularization and patency of grafts remain uncompromised by using a minimal-access approach. Given the paucity of RCTs regarding methods of minimal-access coronary artery revascularization, it is challenging to make any robust recommendations. Part of this comes from the large variation in the nomenclature of the methods of minimal-access coronary artery revascularization and the very slow uptake of minimal-access methods across different surgical units.

## Figures and Tables

**Table 1:** Comparing the different modalities of minimal-access coronary artery surgery [37,38,39,40,41,42].

	MIDCABG	MICS CABG	TECABG/RACABG	HCR
Contra- indications	Absolute: Emergency surgery with haemodynamic compromise Severe pectus excavatum Severe pulmonary disease In TECABG/RACABG, the presence of severe left pleural scarring Relative: Left subclavian artery stenosis Haemodialysis arteriovenous fistula on the patient’s left side Re-do surgery Morbid obesity Severe LV dysfunction Need for right coronary artery graft with no posterior descending or left ventricular branch target Need for circumflex coronary artery graft with no adequate marginal branch target and absence of femoral pulses bilaterally			
Advantages	Avoids the use of CPB	Allows complete revascularization in the presence of three-vessel or diffuse coronary artery disease Allows complete harvest of the LIMA, whether skeletonised or not Allows access to all coronary arteries and their territories Allows proximal anastomoses to be routinely performed	Transthoracic assistance may not be necessary for RACABG if a fourth robotic arm is available Minimal surgical trauma Allows multivessel revascularization Smaller incisions Less pain because no retractor is required for LIMA harvest	Avoids the use of CPB Still obtains the prognostic benefit of LIMA to LAD graft but complete revascularisation of other territories as well through PCIs
Dis- advantages	Restricted to single LIMA to LAD graft Cannot access all coronary artery territories Still requires a thoracotomy, which can be painful Does not lend itself to intramyocardial targets	Difficult to harvest RIMA Reasonable patency rate at 6 months	A long learning curve with higher initial rates of LIMA to LAD anastomosis failure, LIMA injuries, and longer bypass times Access depends on the port position	LIMA to LAD anastomosis failure more common than with standard CABG The use of antithrombotic medications and contrast are required for PCIs very soon before or after a major cardiac procedure More than one major intervention within days of each other

**Table 2:** Summary of major studies (>150 patients) looking at the outcomes of minimal-access coronary revascularisation ((-) means that the data were not reported by the authors).

Authors	Surgical Technique	Patients	Retrospective vs. Prospective	Survival	Follow-Up/Months	Sternotomy Conversion	Peri-Op Stroke	LOS	Number of Grafts	Complete Revascularisation	LIMA–LAD Patency	Repeat Revascularisation
McGinn et al. (2009) [35]	MIDCABG	450	Retrospective	98.7%	1	3.8%	0.4%	5.9 +/− 3.4	2.1 +/− 0.7	95%	-	2.7%
Lapierre et al. (2011) [70]	MIDCABG	150	Retrospective	100.0%	3	6.7%	0.0%	5.0	1.8 +/− 0.7	100%	-	3.3%
Zianku et al. (2015) [71]	MIDCABG	151	Retrospective	99.3%	40.3	2.7%	0.0%	4.5	2.9 +/− 0.5	100%	100.0%	-
Rodriguez et al. (2017) [72]	MIDCABG	306	Retrospective	100.0%	33.6	3.3%	0.0%	5.8 +/− 5.5	1.8 +/− 0.7	93%	-	6.9%
Nambiar et al. (2019) [73]	MIDCABG	940	Retrospective	99.1%	2.9	0.6%	0.2%	3.1 +/− 1.2	3.2	97.90%	99.80%	1.1%
Bonaros et al. (2013) [74]	TECABG	500	Retrospective	99.0%	120	10.0%	9.0%	6.0	-	-	90–95%	-
Weldinger et al. (2014) [75]	TECABG	384	Retrospective	99.2%	60	14.0%	1.8%	7.0	-	-	-	-
Kitahara et al. (2018) [76]	TECABG	263	Retrospective	98.5%	1	3.0%	0.0%	3.5 +/− 2.9	-	-	-	-
Repossini et al. (2013) [77]	HCR with MIDCABG	166	Retrospective	95.8%	54	2.4%	N/A	6.5	-	Functionally complete 100%, anatomically incomplete 16.9%	100% before PCI	7.2%
Halkos et al. (2014) [78]	HCR with RA-MIDCABG	300	Retrospective	98.7%	1	2.0%	1.0%	5.0	-	-	97.60%	4.3%
Puskas et al. (2016) [79]	HCR (variable)	200	Prospective	98.5%	1.5 years	0.5%	0.0%	-	-	75.20%	-	7.0%

## Data Availability

Not applicable.

## Abbreviations

AH-TECABG	Totally Endoscopic Arrested Heart Coronary Artery Bypass Grafting
CABG	Coronary Artery Bypass Grafting
CPB	Cardiopulmonary bypass
HCR	Hybrid Coronary Revascularisation
LAD	Left Anterior Descending Artery
LIMA	Left Internal Mammary Artery
MICS CABG	Minimally Invasive Coronary Artery Bypass Grafting
MIDCABG	Minimally invasive Coronary Artery Bypass Grafting
PA CABG	Port-Access Coronary Artery Bypass Grafting
RACABG	Robotic-Assisted Coronary Artery Bypass Grafting
RATS	Robot-Assisted Thoracoscopic Surgery
RCT	Randomised Control Trial
RIMA	right Internal Mammary Artery
TECABG	Totally Endoscopic Coronary Artery Bypass Grafting
VATS	Video-Assisted Thoracoscopic Surgery
